# Expert consensus on the bone repair strategy for osteoporotic fractures in China

**DOI:** 10.3389/fendo.2022.989648

**Published:** 2022-10-26

**Authors:** Hao Zhang, Yan Hu, Xiao Chen, Sicheng Wang, Liehu Cao, Shiwu Dong, Zhongmin Shi, Yanxi Chen, Liming Xiong, Yunfei Zhang, Dianying Zhang, Baoqing Yu, Wenming Chen, Qining Wang, Peijian Tong, Ximing Liu, Jianzheng Zhang, Qiang Zhou, Feng Niu, Weiguo Yang, Wencai Zhang, Yong Wang, Shijie Chen, Jinpeng Jia, Qiang Yang, Peng Zhang, Yong Zhang, Jun Miao, Kuo Sun, Tao Shen, Bin Yu, Lei Yang, Lei Zhang, Dongliang Wang, Guohui Liu, Yingze Zhang, Jiacan Su

**Affiliations:** ^1^ Institute of Translational Medicine, Shanghai University, Shanghai, China; ^2^ Changhai Hospital, Naval Medical University, Shanghai, China; ^3^ Department of Orthopedics, Shanghai Zhongye Hospital, Shanghai, China; ^4^ Department of Orthopedics, Shanghai Baoshan Luodian Hospital, Shanghai, China; ^5^ Department of Biomedical Materials Science, Army Medical University, Chongqing, China; ^6^ Shanghai Jiao Tong University Affiliated Sixth People’s Hospital, Shanghai, China; ^7^ Zhongshan Hospital, Fudan University, Shanghai, China; ^8^ Union Hospital, Tongji Medical College, Huazhong University of Science and Technology, Wuhan, China; ^9^ Tangdu Hospital, Air Force Medical University, Xi'an, China; ^10^ People’s Hospital, Peking University, Beijing, China; ^11^ Department of Orthopedics, Shanghai Pudong Hospital, Shanghai, China; ^12^ Institute of Biomedical Engineering, Academy for Engineering and Technology, Fudan University, Shanghai, China; ^13^ Department of Advanced Manufacturing and Robotics, College of Engineering, Peking University, Beijing, China; ^14^ Department of Orthopedics, The First Affiliated Hospital of Zhejiang Chinese Medical University, Hangzhou, China; ^15^ Department of Orthopedics, General Hospital of Central Theater Command, Wuhan, China; ^16^ Department of Orthopedic Surgery, People's Liberation Army (PLA), Army General Hospital, Beijing, China; ^17^ Department of Orthopedics, The Third Affiliated Hospital of Chongqing Medical University, Chongqing, China; ^18^ Department of Orthopedics, The First Hospital of Jilin University, Changchun, China; ^19^ Li Ka Shing Faculty of Medicine, Hongkong University, Hong Kong, Hong Kong SAR, China; ^20^ Department of Orthopedics, The Third Affiliated Hospital of Guangzhou University of Traditional Chinese medicine (TCM), Guangzhou, China; ^21^ Department of Orthopedics, Wenzhou Hospital of Integrated Traditional Chinese and Western Medicine, Wenzhou, China; ^22^ Department of Orthopedics, The Third Xiangya Hospital of Central South University, Changsha, China; ^23^ Department of Orthopedics, Chinese People’s Liberation Army General Hospital, Beijing, China; ^24^ Department of Orthopedics, Tianjin Hospital, Tianjin, China; ^25^ Department of Orthopedics, Shandong Province Hospital, Jinan, China; ^26^ Department of Orthopedics, The Second Affiliated Hospital of Nanchang University, Nanchang, China; ^27^ Department of Orthopedics, Peking Union Medical College Hospital, Beijing, China; ^28^ Second Affiliated Hospital and Yuying Children’s Hospital of Wenzhou Medical University, Wenzhou, China; ^29^ Shuguang Hospital, Shanghai University of Traditional Chinese Medicine, Shanghai, China; ^30^ Xinhua Hospital, School of Medicine, Shanghai Jiao Tong University, Shanghai, China; ^31^ Department of Orthopedics, Third Hospital of Hebei Medical University, Shijiazhuang, China

**Keywords:** osteoporosis, fracture, bone repair, expert consensus, biomaterials

## Abstract

Osteoporotic fractures, also known as fragility fractures, are prevalent in the elderly and bring tremendous social burdens. Poor bone quality, weak repair capacity, instability, and high failure rate of internal fixation are main characteristics of osteoporotic fractures. Osteoporotic bone defects are common and need to be repaired by appropriate materials. Proximal humerus, distal radius, tibia plateau, calcaneus, and spine are common osteoporotic fractures with bone defect. Here, the consensus from the Osteoporosis Group of Chinese Orthopaedic Association concentrates on the epidemiology, characters, and management strategies of common osteoporotic fractures with bone defect to standardize clinical practice in bone repair of osteoporotic fractures.

## Introduction

Osteoporosis is a systemic bone disease characterized by reduced bone mass, bone microstructure damage, leading to increased bone fragility and susceptibility to fracture (1). The latest epidemiological data showed that the prevalence of osteoporosis in individuals of 40–49 years in China was 2.4% in men and 4.3% in women, the prevalence of osteoporosis in people aged 50–59 years was 4.6% in men and 16.4% in women, the prevalence of osteoporosis in populations 60–69 years reached 5.4% in men and 37.1% in women, and the prevalence of osteoporosis in populations 70–79 years reached 12.3% in men and 67.5% in women (2). The highest prevalence of osteoporosis reached to 79.8% in women older than 85 years old (3).

Osteoporotic fractures, also known as fragility fractures, refer to fractures that occur after minor trauma or in daily activities and are commonly found in the vertebrae, hip, distal forearm, proximal humerus, and distal tibia in elderly (4, 5). Aging of the world populations increased the incidence of osteoporotic fractures in recent years. The prevalence of vertebral fractures in China is about 15% in women over 50 years old and 36.6% in women over 80 years old. The incidence of hip fractures in elder over 50 years old was 83/100,000 for men and 80/100,000 for women in 1990–1992 and 129/100,000 for men and 229/100,000 for women in 2002–2006 (6). It is estimated that the number of osteoporotic fractures in China will be 4.83 million in 2035 and will reach 5.99 million in 2050 (7).

The basic changes in bone structure of osteoporosis are loss of bone mass and reduction of bone density. Specifically, osteoporosis patients are manifested by thinning of the cortex bone, sparseness of cancellous bone, and increased bone fragility. It is difficult to obtain stable compression and fixation of fracture with conventional internal fixation. The implant cannot firmly integrate with bone, which may lead to internal fixation failure (8). Poor osteointegration combined with molecular and cellular defects of osteoporosis increases the risk of internal fixation failure and bone healing (9). Osteoporotic fractures are often accompanied by bone defects due to changes in bone microarchitecture, decreasing deposits of bone mineral and bone matrix components, sparseness of bone trabecular, decreased bone strength, and increased bone fragility (10, 11). To achieve good clinical outcomes, bone implants or bone substitutes are required for osteoporotic bone defect repair and internal fixation augmentation. Appropriate bone grafting provides sufficient biomechanical support for fracture healing and bone repair. In addition, the application of memory alloy-fixation system can improve clinical outcomes in fractures with large bone defect, articular surface collapse, and bone non-union (12–14).

In order to provide a suggestion for clinical treatment of osteoporotic fracture, this consensus is developed by the Osteoporosis Group of the Chinese Orthopaedic Association, in collaboration with the Orthopaedic Specialist Committee of the Chinese Society of Gerontology and Geriatrics, the Traumatic Orthopaedics and Multiple Injuries Group of the Emergency Resuscitation Committee of the Chinese Medical Association, and the Osteoporosis Committee of the Shanghai Society of Integrated Traditional Chinese and Western Medicine.

## Bone repair materials in osteoporotic fractures

High porosity and low strength of cancellous bone lead to failure of internal fixation in osteoporosis patients (10). Bone grafting can fill the bone defect to improve biomechanical properties. Current bone repair materials should have at least one of these two roles: (1) bone conductive role, which provides mechanical stability and enhances osteointegration of the implant or (2) bone inducible role, which enhances bone repair by inducing bone remodeling. Clinical bone repair materials are autologous bone, allogeneic bone, and artificial bone.

### Autologous bone

Autologous bone graft is the “gold standard” for the treatment of bone defects. Cortical bone can be selected for structural grafting to increase fracture stability. Cancellous bone can be used to fill the bone defect to facilitate fracture healing. Autologous cortical bone grafts have excellent structural integrity and can provide mechanical support in the early stages of fracture healing (15). For osteoporotic proximal humeral fractures, autogenous fibular segment graft combined with locking plate fixation can enhance the support of the internal fixation, against varus stress, reduce the risk of internal fixation failure and humeral head necrosis (16–18). The large surface area of autogenous cancellous bone grafts facilitates vessel reconstruction and bone conduction. Meanwhile, autologous cancellous bone is rich in mesenchymal stem cells (MSCs) and bone inductive factors that promote osteogenesis (19). However, autologous bone still has several disadvantages. Sources of autologous bone are limited. Fibula and iliac bone are common sources of autogenous bone grafting. Trauma, infection, or former bone graft history of donor site may restrict autologous bone application. Obtaining autologous bone is an invasive procedure with risks associated with surgery such as bleeding and infection. These potential complications may cause secondary damage to patients.

### Allogeneic bone

Allogeneic bone is a suitable substitute for autologous bone. Allogeneic bone can be obtained from living or non-living donors and preserved in bone tissue bank. Allogeneic cancellous bone grafts are mainly used in spinal fusion enhancement and filling bone defects in patients with osteoporosis. Cortical allografts are primarily used for vertebroplasty to fill bone defects that require immediate loading. Demineralized bone matrix is also a kind of bone graft material for spinal fusion, bone non-union, and bone defects. The integral property of allogeneic bone is similar to autologous bone, triggers endochondral ossification, and, eventually, forms new bone at the implantation site (20). The disadvantages of allogenic are immune rejection and lack of bioactivity. There is a risk of immune rejection of allogeneic bone grafts, which may lead to local redness, swelling even bone resorption. The lack of bioactivity of allogeneic leads a longer duration of bone healing. Those disadvantages are not unacceptable compared with the wide source of homogeneous allogeneic bone and the unrestricted dosage.

### Artificial bone material

Calcium sulfate grafts are absorbable synthetic bone substitutes, which could be resorbed within 1–3 months faster than bone grafts (21–23). Calcium phosphate (CaP) ceramics are a family of calcium salt compounds composed of varying proportions of calcium ions and organic phosphates. CaP ceramics have applied as an absorbable ceramic with good bone conductivity (24–27).

Bone cement has been used in the treatment of compressive osteoporotic vertebral fractures. However, the risk of complications caused by bone cement still exists. Indications of bone cement application should be controlled. In addition, the procedure of bone cement should be noticed to prevent leakage. Polymethylmethacrylate (PMMA) bone cement is widely used in clinical practice. PMMA bone cement can stabilize the injured vertebral body rapidly and relieve patients’ symptoms. However, PMMA has no bone conductive property and cannot be integrated within the host bone. PMMA cannot conduce adhesion and growth of bone cells after injected into fracture sites. High modulus and stiffness of PMMA can easily lead to local microfracture and compression fractures of adjacent vertebrae (28). Calcium phosphate cement (CPC) is a white powder and a good substitute for bone grafts. CPC is widely used to fill bone defects in fragility fractures. The remodeling process of CPC occurs at the bone-cement interface where deposition of new bone and resorption of CPC occurred simultaneously (29). Zinc, magnesium, copper, and other metal ion can be added in artificial bone materials to promote bone repair in a delicate concentration (30–34). Bioactive composite also showed great prospect in application of bone repair (35–37). Bioactive materials such as biocompatible hydrogel or materials with bioactive factors such as BMP-2, MMP-cleavable peptides were reported in treating large bone defect with good outcomes (38–40). The bioactive materials that carry bioactive factors to create bone organoid may be a new research direction in bone repair (41).

### Bone-targeting biomaterials

Bone-targeting materials are mainly divided into two categories: matrix-targeted materials and cell-targeted materials (42). Inorganic hydroxyapatite (HA) is the main component of bone matrix. Matrix-targeted materials select HA high-affinity substances as drugs or drug carriers, mainly include tetracycline and bisphosphonates (43, 44). The cellular components of bone tissue include MSCs, osteoblasts, osteoclasts, and adipocytes (45). Complex functions and the interaction of bone cells need that the drug delivery system has precise cells targeted ability (46, 47). Advances in cell-targeted materials research used high-cell affinity peptides and nucleic acids as targeting components and growth factors as drug components (48). Recently, several studies reported that several exosomes from special origin are highly bone-targeting *in vivo* and have high drug delivery potential (49–52). Reactive Oxygen Species (ROS) appear in many aging diseases and can be a target in osteoporosis (53). There are many drugs that have bone-targeted function, including many small molecules from traditional Chinese drugs, which were reported could improve osteogenesis in fracture (53–55).

## Bone augmentation strategies for common osteoporotic fractures

### Proximal humerus fracture

The proximal humerus is a common site for osteoporotic fractures in the elderly. Conservative treatment is the first choice and gold standard for proximal humerus fractures with insignificant fracture displacement (lower than 1 cm). For large displacement fracture, the presence of significant bone loss in metaphysis of the proximal humerus fracture often results in fracture displacement and internal fixation failure after surgery, impedes early exercise of shoulder (56).

Fibular graft and calcium phosphate bone cement are suitable bone strengthen materials for different types of proximal humerus fracture. For varus proximal humerus fractures with defect of medial support, fibular bone grafting of fibular segment is feasible to support the humeral medial screw. Fibular segment graft can fill the bone defect and achieve good mechanical support of the medial cortex, reduce micro-movement of screws, strengthen the stability of the plate screw system, and reduce the incidence of postoperative complication (57, 58). In addition, allogeneic iliac bone and femoral head grafts can also increase bone volume and provide cortical enhancement of internal fixation but are not preferred (59). However, homogeneous fibular segments are of limited origin and are only indicated for severely comminuted proximal humeral fractures that lack medial support. Vascularized fibular graft can also promote bone healing but make second damage to patients so is not preferred.

For valgus-impacted fractures, bone defects occur at the lateral wall of the proximal humerus. The application of calcium phosphate bone cement technique during surgery can increase the strength of the bone and improve the local mechanical strength (60). Injectable calcium phosphate bone cement has certain advantages during operation. Whether to use CPC should consider the degree of bone defect and degree of osteoporosis. CPC and PMMA bone cement can be used as a bone augmentation for most osteoporotic proximal humerus fractures, but its mechanical strength is weaker than that of bone graft. Therefore, for proximal humeral fractures lacking support in the non-medial wall, bone cement injection can effectively enhance the treatment outcome. Proximal humeral fractures lacking medial support is the indication for bone grafting. Fibular graft is recommended, and other methods can be selected depending on the operative situations. Local bone substitute filling plays a positive role in treating valgus-impacted proximal humeral fractures.

### Distal radius fracture

Distal radius fracture is the second prevalent osteoporotic fracture (61). Non-operative treatment including closed reduction and immobilize with splint and cast is recommended for a majority of distal radius fracture (62). Osteoporotic distal radius fractures have bone defects, articular surface collapse, fracture displacement after reduction, or secondary fracture need surgical intervention. Autologous iliac cancellous bone filling and inlay support with cortical bone can achieve structural reconstruction and prevent distal articular surface collapse. Stable maintenance of reduction after bone graft prevents loss of distal radius height and fracture re-displacement (63). Homogeneous bone and artificial bone are also an optional selection and avoid second damage (62). CaP injection combined with volar locking plate fixation of distal radius fracture is still controversial and is not preferred in this recommendation (64, 65). Autogenous iliac bone is ideal for effectively restoring the height of the distal radius, providing support to the collapsed cartilage surfaces, and increasing the stability of the internal fixation. Allograft bone and artificial bone may also be an alternative option.

### Tibial plateau fracture

Ostesoporotic tibial plateau fracture is not common in elderly. Schatzker types I, II, and III fractures are main fracture type because of low-energy trauma (66). Conservative treatment through cast or orthosis is indicated for patients with small or non-displacement. For patients with significant displacement and acceptable soft tissue condition, plate fixation can promote functional rehabilitation under-weight bearing (67). Although high-energy tibial plateau fracture is not common in osteoporosis patient, late repair and total knee arthroplasty are recommended to protect soft tissue and restore knee function. The goals of surgical treatment are joint surface reconstruction and strong internal fixation. Bone graft assisted locking plate internal fixation has shown good outcome in the treatment of tibial plateau fractures (68). For Schatzker II–VI type tibial plateau fractures with articular surface collapse greater than 5 mm, calcium phosphate or calcium sulfate injected artificial bone can be used to fill the bone defect to prevent postoperative articular surface collapse and reduce the occurrence of traumatic osteoarthritis. Sufficient artificial bone graft can maintain anatomical reduction and support internal fixation with definite clinical results. However, it is not clear whether artificial bone resorption is coupled with bone formation and whether a bone defect will form after resorption. Therefore, further research is needed. Bone graft combines with locking plate internal fixation therapy is still the mainstream in osteoporotic tibial plateau fractures treatment. Calcium phosphate or calcium sulfate injectable artificial bone can be used as a bone repair material for tibial plateau fractures with large articular surface collapse.

### Pilon fracture

The collapse of the distal tibial articular surface in pilon fractures is caused by axial force, and intra-articular damage is severe in patients with osteoporosis (69). The four classical principles of pilon fractures treatment are as follows: restoration of fibular length, reconstruction of tibial articular surface, autologous bone grafting, and application of buttress plates (70). Autologous iliac bone graft or allogeneic bone graft can be used in the treatment of pilon fracture to enhance the mechanical support of the articular surface to increase stability of fracture sites. Bone graft can promote fracture healing and prevent the occurrence of late articular surface collapse. Autogenous iliac bone or allograft bone can be chosen as bone grafting material in treatment of pilon fractures.

### Calcaneus fracture

It is still controversial whether bone graft is needed in calcaneus fracture. For Sanders’ type II and above calcaneus fracture, bone defects larger than 2 cm^3^, articular surface collapse large than 2 mm or difficult to maintain articular surface, bone graft is beneficial if soft tissue conditions allow. Bone graft permits early postoperative weight-bearing rehabilitation and helps to maintain articular surface stability (71, 72). Because calcaneus infection is a disaster for patient, it is generally considered safer to use autologous bone or allogeneic bone (73). The necessity for bone graft in calcaneus fractures remains controversial, and decision is based on the degree of articular surface collapse and bone defect.

### Vertebral fracture

Osteoporotic Vertebral Compression Fracture (OVCF) happened within 3 months with significant pain, and an intact posterior wall of vertebra should be treated with vertebroplasty. Percutaneous vertebroplasty (PVP) and percutaneous kyphoplasty (PKP) are main vertebroplasty in clinic; both of them can restore the height and strength of the compressed vertebral body, improve spinal stability, prevent vertebral collapse, relieve pain, and improve spinal function. PKP has advantages in reducing the occurrence of cement leakage, restoring the height of the vertebral body, and correcting spinal deformity. In severe OVCF with vertebral height less than 1/3 of the original height, PKP is superior to PVP in restoring normal vertebral angle and height (73, 74). There is no consensus on how to choose between these two techniques; decision should be made according to preoperative condition and radiology image (75, 76). The amount of bone cement should be limited to 2–5 ml. Excessive bone cement may increase the risk of cement leakage, but long-term benefits are limited (77, 78). In clinical practice, the amount of bone cement should consider the size of the vertebral body, operative fluoroscopy, and the operator’s experiences.

For OVCF with significant spinal cord injury, pedicle screws internal fixation with decompression and reduction is an option in patients without contraindications. There are no uniform criteria whether to use cement reinforcement in pedicle screw fixation. If the bone quality is poor during operative evaluation or screw loosens after nail insertion, cement strengthening techniques are indicated. The reinforced segment may choose 1–2 screws either in cephalad or caudal, and the whether to reinforce screws in the intermediate segment may be decided by operative condition (75, 79).

For most patients with OVCF, PVP or PKP can achieve similar outcomes, but PKP is more suitable for severe compression fractures. Strict operation is required to avoid the risk of cement leakage. Bone cement strengthening technique can be used as an important technique in OVCF internal fixation, which can help reduce incidence of internal fixation failure and loss of reduction.

## Rehabilitation aids in osteoporotic fractures

Rehabilitation aids can be a non-operative treatment for osteoporotic fractures and accelerate postoperative rehabilitation. Early exercise and weight-bearing under the protection of aids are important for fracture healing and avoiding deterioration of osteoporosis.

Osteoporotic distal radius fractures need more time to recover and may lead to long-term functional disorders (80). The functional disorders have severe damage to ability and quality of life. Static stretch splints and dynamic stretch splints can lengthen soft tissues and restore range of motion to contracted joints. Those splints can be used to treat persistent wrist stiffness and prevent bone loss after distal radius fractures and are effective when used in the early stage of rehabilitation (81).

Rehabilitation aids have been widely used in the treatment of osteoporotic foot and ankle fractures. The intrepid dynamic exoskeletal orthosis (IDEO) is a foot and ankle orthosis with energy storage-redistribution function. IDEO is originally designed for the rehabilitation of soldiers with complex lower extremity trauma to cure gait disorders. IDEO is also beneficial for patients with post-traumatic osteoarthritis, mild paralysis, and muscle atrophy (82). Studies have shown that IDEO improves walking speed in patients after pilon fracture and may be helpful for patients with high demand of activity (83). Calcaneus orthosis can be a non-operative treatment of calcaneus fractures without displacement. Full weight bearing can be achieved with the protection of the calcaneus orthosis. The pressure pad can be adjusted to gradually increase the weight bearing on the foot that facilitates early weight bearing and rehabilitation (84). In displaced osteoporotic calcaneus fractures, orthosis can also accelerate postoperative recovery.

The thoracolumbar orthosis provides rigid support and increases intra-abdominal pressure. Orthosis provides a semi-rigid cylindrical support around the spine and distributing the load on the spine. Clinical trials have demonstrated that thoracolumbar orthoses significantly increase trunk muscle strength, improve lung function, reduce kyphosis, and pain in patients with OVCF (84–86). Traditional rigid spinal orthoses are limited due to trunk muscle atrophy and restriction of breath and harmful for patients with osteoporosis (87). Dynamic thoracolumbar orthoses have a lower degree of immobilization based on the biofeedback activation of the low-back muscles and reported good clinical results (88). Although the application of thoracolumbar orthoses as a treatment in vertebral fracture is still contentious, it could be used as an aid in post-operative rehabilitation.

**Consensus:** Dynamic and static stretch splints are effective for early rehabilitation of distal radius fractures; foot and ankle orthoses have been widely used for foot and ankle osteoporotic fractures, facilitating early weight bearing and rehabilitation training; and dynamic thoracolumbar orthoses can help patients with osteoporotic thoracolumbar fractures to increase muscle strength and reduce pain.

## Statement

This consensus is not a clinical treatment standard for osteoporotic fractures in the elderly but only an academic guideline recommendation. Under the constraints of individual patient and actual clinical conditions, the clinical treatment plan varies from person to person. With the development of medical technology, some parts of this consensus will be further improved.

## Author contributions

HZ, YH, XC and SW contributed equally to this work. All authors contributed to the article and approved the submitted version.

## Funding

National Key Research and Development Program (2018YFC2001500); National Natural Science Foundation of China Major Research Program Key Project (91749204); National Natural Science Foundation of China (81771491); Shanghai Health System Excellent Discipline Leader Program (2017BR011)

## Conflict of interest

The authors declare that the research was conducted in the absence of any commercial or financial relationships that could be construed as a potential conflict of interest.

The reviewer MY declared a shared parent affiliation with the authors ZS and DW at the time of review.

## Publisher’s note

All claims expressed in this article are solely those of the authors and do not necessarily represent those of their affiliated organizations, or those of the publisher, the editors and the reviewers. Any product that may be evaluated in this article, or claim that may be made by its manufacturer, is not guaranteed or endorsed by the publisher.
